# Efficient mRNA Delivery with mRNA Lipoplexes Prepared Using a Modified Ethanol Injection Method

**DOI:** 10.3390/pharmaceutics15041141

**Published:** 2023-04-04

**Authors:** Min Tang, Ayane Sagawa, Nodoka Inoue, Satomi Torii, Kana Tomita, Yoshiyuki Hattori

**Affiliations:** Department of Molecular Pharmaceutics, Hoshi University, 2-4-41 Ebara, Shinagawa, Tokyo 142-8501, Japan

**Keywords:** mRNA, lipoplex, delivery, mRNA vaccine

## Abstract

Messenger RNA (mRNA)-based therapies are a novel class of therapeutics used in vaccination and protein replacement therapies for monogenic diseases. Previously, we developed a modified ethanol injection (MEI) method for small interfering RNA (siRNA) transfection, in which cationic liposome/siRNA complexes (siRNA lipoplexes) were prepared by mixing a lipid-ethanol solution with a siRNA solution. In this study, we applied the MEI method to prepare mRNA lipoplexes and evaluated the in vitro and in vivo protein expression efficiencies. We selected six cationic lipids and three neutral helper lipids to generate 18 mRNA lipoplexes. These were composed of cationic lipids, neutral helper lipids, and polyethylene glycol-cholesteryl ether (PEG-Chol). Among them, mRNA lipoplexes containing *N*-hexadecyl-*N*,*N*-dimethylhexadecan-1-aminium bromide (DC-1-16) or 11-((1,3-bis(dodecanoyloxy)-2-((dodecanoyloxy)methyl) propan-2-yl) amino)-*N*,*N*,*N*-trimethyl-11-oxoundecan-1-aminium bromide (TC-1-12) with 1,2-dioleoyl-*sn*-glycero-3-phosphoethanolamine (DOPE) and PEG-Chol exhibited high protein expression in cells. Furthermore, mRNA lipoplexes composed of DC-1-16, DOPE, and PEG-Chol exhibited high protein expression in the lungs and spleen of mice after systemic injection and induced high antigen-specific IgG1 levels upon immunization. These results suggest that the MEI method can potentially increase the efficiency of mRNA transfection, both in vitro and in vivo.

## 1. Introduction

Messenger RNA (mRNA) is a single-stranded molecule that encodes a protein synthesis sequence that can be translated into a protein by the ribosome. mRNA has become one of the most promising therapeutic modalities because it enables the immediate and efficient expression of therapeutic proteins in cells [1]. The therapeutic use of mRNA has several advantages; mRNAs only need to be delivered to the cytoplasm for protein translation [2], they cannot be integrated into chromosomal DNA [3], and they can be easily produced by in vitro transcription [4]. The most advanced application of mRNA therapeutics is in vaccinations against viral infections [5,6]. mRNA vaccines encode only the surface antigens of viral proteins and can elicit potent immunological responses against these antigens upon translation in the cells. In the application of mRNA vaccines for cancer immunotherapy, mRNA encodes tumor antigens or immunomodulatory molecules that trigger an anti-tumor response [7]. The injection routes of mRNA cancer vaccines include subcutaneous, intramuscular, and intravenous injections [8,9]. However, mRNAs cannot diffuse through cellular membranes owing to their hydrophilic nature and negative charge, and are susceptible to enzymatic degradation by serum endonucleases [10]; therefore, efficient mRNA delivery systems are needed [11,12,13].

Several carriers for mRNA delivery have been reported, including cationic liposomes [14,15,16], lipid nanoparticles (LNPs) [17,18,19], and cationic polymers [20]. Among them, LNPs containing cationic ionizable lipids are the most advanced mRNA delivery systems [18,21]. LNPs expressing the SARS-CoV-2 spike (S) protein have been developed as coronavirus disease 2019 (COVID-19) vaccines by Pfizer–BioNTech (Mainz, Germany) (Comirnaty^®^) [22] and Moderna (Cambridge, MA, USA) (Spikevax^®^) [23]. These LNPs consist of four main lipid components: phospholipids, cholesterol, PEG-lipids, and ionizable cationic lipids. The most important components of LNPs are ionizable cationic lipids, which are designed with a pKa below 7 to regulate the encapsulation of mRNA into LNPs at acidic pHs and stabilize systemic circulation at a physiological pH [24]. Recently, microfluidic mixing devices have been used to prepare LNPs [25], in which lipids dissolved in an organic solvent and mRNAs in an aqueous solution are mixed rapidly on a microfluidic chip to produce homogenous particles with a narrow size distribution and high mRNA encapsulation efficiency; however, the preparation of these mRNA-LNPs requires special equipment.

Previously, we developed a simple technique, known as the modified ethanol injection (MEI) method, for preparing small interfering RNA (siRNA) lipoplexes by rapidly mixing a lipid-ethanol solution with phosphate-buffered saline (PBS) containing siRNAs [26]. We demonstrated that the siRNA lipoplexes prepared using this method exhibited high gene knockdown activity both in vitro and in vivo. Therefore, in the present study, we used the MEI method to prepare mRNA lipoplexes. We selected 6 cationic lipids and 3 neutral helper lipids to generate 18 mRNA lipoplexes composed of each cationic lipid with a neutral helper lipid and polyethylene glycol cholesteryl ether (PEG-Chol) by simply mixing a lipid-ethanol solution and PBS containing mRNAs. Furthermore, we evaluated the in vitro and in vivo protein expression efficiencies of these mRNA lipoplexes.

## 2. Materials and Methods

### 2.1. Materials

1,2-Dioleoyl-3-trimethylammonium-propane methyl sulfate salt (DOTAP) was obtained from Avanti Polar Lipids, Inc. (Alabaster, AL, USA). Dimethyldioctadecylammonium bromide (DDAB, DC-1-18), *N*-hexadecyl-*N*,*N*-dimethylhexadecan-1-aminium bromide (DC-1-16), *N*,*N*-dimethyl-*N*-tetradecyltetradecan-1-aminium bromide (DC-1-14), 2-(bis(2-(tetradecanoyloxy)ethyl)amino)-*N*,*N*,*N*-trimethyl-2-oxoethan-1-aminium chloride (DC-6-14), and 11-((1,3-bis(dodecanoyloxy)-2-((dodecanoyloxy)methyl) propan-2-yl) amino)-*N*,*N*,*N*-trimethyl-11-oxoundecan-1-aminium bromide (TC-1-12) were obtained from Sogo Pharmaceutical Co., Ltd. (Tokyo, Japan). Polyethylene glycol-cholesteryl ether (PEG-Chol, mean MW: 2000), 1,2-dioleoyl-*sn*-glycero-3-phosphoethanolamine (DOPE, COATSOME^®^ ME-8181), and 1,2-dioleoyl-*sn*-glycero-3-phosphocholine (DOPC, COATSOME^®^ MC-8181) were purchased from NOF Co., Ltd. (Tokyo, Japan). Cholesterol (Chol) was purchased from FUJIFILM Wako Pure Chemical Industries, Ltd. (Osaka, Japan). All other chemicals were of the highest available grade.

### 2.2. mRNAs

Firefly luciferase mRNA (FLuc mRNA, 1929 nucleotides, CleanCap^®^ FLuc mRNA), FLuc mRNA modified with 5-methoxyuridine (FLuc mRNA (5moU), 1929 nucleotides, CleanCap^®^ FLuc mRNA (5moU)), enhanced green fluorescent protein mRNA (EGFP mRNA, 996 nucleotides, CleanCap^®^ EGFP mRNA), cyanine 5 (Cy5)-labeled FLuc mRNA modified with 5-methoxyuridine (Cy5-mRNA (5moU), CleanCap^®^ Cyanine 5 FLuc mRNA (5moU)), and ovalbumin mRNA modified with 5-methoxyuridine (OVA mRNA, 1437 nucleotides, CleanCap^®^ OVA (5moU) mRNA) were obtained from TriLink Biotechnologies (CA, USA). EZCap^TM^ Cyanine 5 FLuc mRNA (5moU) (Cy5-mRNA (5moU), 1921 nucleotides) was obtained from ApexBio Technology LLC (Boston, MA, USA).

### 2.3. Cell Culture

Human cervical carcinoma (HeLa) cells were obtained from the European Collection of Authenticated Cell Cultures (ECACC, Wiltshire, UK). Human lung adenocarcinoma (A549) cells were gifted by OncoTherapy Science Inc. (Tokyo, Japan). Human prostate carcinoma (PC-3) cells were supplied by the Cell Resource Center for Biomedical Research, Tohoku University (Miyagi, Japan).

The cells were maintained in appropriate growth media in an incubator at 37 °C and 5% CO_2_ humidified atmosphere. HeLa cells were grown in Eagle’s Minimum Essential Medium (EMEM, FUJIFILM Wako Pure Chemical Industries, Ltd.) supplemented with 10% heat-inactivated fetal bovine serum (FBS, Thermo Fisher Scientific, Inc., Rockford, IL, USA) and 100 μg/mL kanamycin while A549 and PC-3 cells were cultured in Roswell Park Memorial Institute (RPMI)-1640 medium (FUJIFILM Wako Pure Chemical Industries, Ltd.) supplemented with 10% heat-inactivated FBS and 100 μg/mL kanamycin.

### 2.4. Preparation of mRNA Lipoplexes for In Vitro Transfection

A lipid-ethanol solution was prepared by dissolving cationic lipid (DOTAP, DDAB, DC-1-16, DC-1-14, DC-6-14, or TC-1-12), neutral helper lipid (DOPE, DOPC, or Chol), and PEG-Chol at a molar ratio of 49.5:49.5:1 in ethanol (Table 1) (2 mg/mL for cationic lipids; for example, 2 mg TC-1-12, 1.53 mg DOPE, and 0.08 mg PEG-Chol were dissolved in 1 mL ethanol) as previously reported [26]. For in vitro transfection, 0.5 μL of 1 mg/mL mRNA solution was transferred into a 1.5 mL tube containing 100 μL of PBS (pH 7.4), and the obtained solution was rapidly added to the lipid-ethanol solution (2.4 μL, 1.6 μL, 1.8 μL, 2.0 μL, 2.1 μL, and 3 μL for DOTAP, DC-1-14, DC-1-16, DDAB, DC-6-14, and TC-1-12 formulation) in another 1.5 mL tube at a charge ratio (+:–) of 4:1, based on previous reports [27,28]; subsequently, the mixture was vortex-mixed for 10 s. 

### 2.5. Size and ζ-Potential Measurements of mRNA Lipoplexes

The particle size distribution, polydispersity index (PDI), and ζ-potentials of FLuc mRNA lipoplexes were measured using an ELS-Z2 light-scattering photometer (Otsuka Electronics Co., Ltd., Osaka, Japan) as previously reported [29]. 

### 2.6. Evaluation of Luciferase Expression in Cells after Transfection with FLuc mRNA Lipoplexes

HeLa, A549, and PC-3 cells were seeded into 12-well culture plates (1 × 10^5^ cells/well). After 24 h of incubation, mRNA lipoplexes with 0.5 μg Fluc mRNA were diluted with a culture medium containing 10% FBS (final concentration: 0.5 μg/mL mRNA and 0.18–0.30% ethanol) and added to the cells. Twenty-four hours after transfection, luciferase activity (counts per second (cps)/μg protein) was measured, as previously reported [29].

### 2.7. Evaluation of EGFP Expression in Cells after Transfection with EGFP mRNA Lipoplexes

HeLa cells were seeded into 12-well culture plates (1 × 10^5^ cells/well). After 24 h of incubation, mRNA lipoplexes with 0.5 μg EGFP mRNA in 1 mL of culture medium were added to the cells (final mRNA concentration of 0.5 μg/mL). Twenty-four hours after transfection, the cells were washed twice with PBS and fixed with 10% formalin neutral buffer solution (Mildform^®^ 10N, FUJIFILM Wako Pure Chemical Industries, Ltd.) for 10 min at room temperature. EGFP expression in the cells was observed using a fluorescence microscope (Eclipse TS100-F, Nikon, Tokyo, Japan).

### 2.8. Cellular Uptake of mRNA Lipoplexes

HeLa cells were seeded into 12-well culture plates (1 × 10^5^ cells/well). After 24 h of incubation, mRNA lipoplexes with 0.5 μg CleanCap^®^ Cy5-mRNA in 1 mL of culture medium were added to the cells (final concentrations of 0.5 μg/mL mRNA). Three hours after the transfection, cells were washed twice with PBS and fixed with 10% formalin neutral buffer solution for 10 min at room temperature. The localization of Cy5-mRNA was observed using a fluorescent microscope.

### 2.9. Cytotoxicity of mRNA Lipoplexes

mRNA lipoplexes with 0.05 μg of Fluc mRNA in 100 μL of culture medium (0.5 μg/mL mRNA) were added to HeLa, A549, or PC-3 cells at 50% confluency in 96-well plates. After 24 h of incubation, the number of viable cells was determined using the Cell Counting Kit-8 (Dojindo Laboratories, Inc., Kumamoto, Japan), as previously reported [28].

### 2.10. Preparation of mRNA Lipoplexes for In Vivo Transfection

A lipid-ethanol solution was prepared by dissolving cationic lipids (DC-1-16 or TC-1-12), DOPE, and PEG-Chol at a molar ratio of 49.5:49.5:1 in ethanol (10 mg/mL for cationic lipids; for example, 10 mg DC-1-16, 12.9 mg DOPE, and 0.65 mg PEG-Chol were dissolved in 1 mL ethanol). For injection of mRNA lipoplexes into mice, 5, 10, and 20 μL of 1 mg/mL mRNA solution were transferred into 50, 100, and 200 μL of PBS (pH 7.4), respectively, in a 1.5 mL tube. The obtained solutions were rapidly added to the lipid-ethanol solution (3.6 μL, 7.2 μL, and 14.3 μL for LP-DC-1-16 lipoplexes with 5, 10, and 20 μg mRNA, respectively, and 6.1 μL, 12.1 μL, and 24.2 μL for LP-TC-1-12 lipoplexes with 5, 10, and 20 μg mRNA, respectively) in another 1.5 mL tube at a charge ratio (+:–) of 4:1; subsequently, the mixture was vortex-mixed for 10 s.

### 2.11. Biodistribution of mRNA after Injection of mRNA Lipoplexes into Mice

All animal experiments were conducted in accordance with the ‘Guide for the Care and Use of Laboratory Animals’ adopted by the Institutional Animal Care and Use Committee of Hoshi University (Tokyo, Japan). Ethical approval was obtained from the Institutional Animal Care and Use Committee of Hoshi University (permission number: P22-016).

Female BALB/c mice (8-week-old; Sankyo Labo Service Corp. Inc., Tokyo, Japan) were housed under standard laboratory conditions (12/12 h light/dark cycle, lights on from 08:00 a.m. to 08:00 p.m.; 24 °C, humidity of 55%), with access to food and water *ad libitum*. mRNA lipoplexes, such as 5 μg of CleanCap^®^ Cy5-mRNA and 10 μg of EZCap^TM^ Cy5-mRNA, were administered by intramuscular (IM) and intravenous (IV) injections, respectively, into these mice. After 1 or 24 h of IM or IV injection, respectively, the mice were humanely sacrificed, and Cy5 fluorescence images of the lungs, heart, liver, spleen, and kidneys were acquired using a NightOWL LB981 NC100 system (Berthold Technologies, Bad Wildbad, Germany) as previously reported [28]. 

### 2.12. Luciferase Expression after Injection of mRNA Lipoplexes into Mice

mRNA lipoplexes with 5 and 20 μg FLuc mRNA (5moU) were administered intramuscularly and intravenously, respectively, into female BALB/c mice (8 weeks old). At 4 or 24-h post-injection of mRNA lipoplexes, mice were sacrificed by cervical dislocation, and tissues were removed for analysis. Three microliters of ice-cold luciferase cell culture lysis reagent (Promega Co., Madison, WI, USA) per 1 mg of tissue was added and homogenized immediately. The homogenized samples were centrifuged at 15,000 rpm for 3 min at 4 °C. Aliquots of 10 μL of the supernatant were mixed with 50 μL of PicaGene MelioraStar-LT Luminescence Reagent (Toyo Ink Co., Ltd., Tokyo, Japan), and counts per second (cps) were measured using a chemoluminometer (ARVO X2; Perkin Elmer Co., Ltd., Waltham, MA, USA) as previously reported [28]. The protein concentration of each supernatant was determined using a bicinchoninic acid (BCA) reagent (Pierce^™^ BCA Protein Assay kit; Thermo Fisher Scientific, Inc.), and luciferase activity was calculated as cps/mg protein.

### 2.13. Measurement of Ovalbumin (OVA) Antibody in Mice Post OVA mRNA Injection

LP-DC-1-16/DOPE lipoplexes with 20 μg FLuc mRNA (5moU) or OVA mRNA (5moU) were systemically injected into female BALB/c mice (8 weeks old) on days 0 and 14. Blood samples were collected from immunized mice on day 28, and the serum was separated by centrifugation. The OVA-specific IgG1 antibody titer (mU/mL) in the serum was quantified using the LBIS mouse anti-OVA-IgG1 ELISA Kit (Fujifilm Wako Shibayagi Corp., Gunma, Japan), according to the manufacturer’s instructions.

### 2.14. Statistical Analysis

Statistical analysis was performed with the unpaired Student’s t-test or one-way ANOVA, followed by Tukey’s post hoc test, using GraphPad Prism (version 4.0; GraphPad Software Inc., San Diego, CA, USA). A p value of 0.05 or less was considered significant. 

## 3. Results

### 3.1. Size of mRNA Lipoplexes 

**Figure 1:**
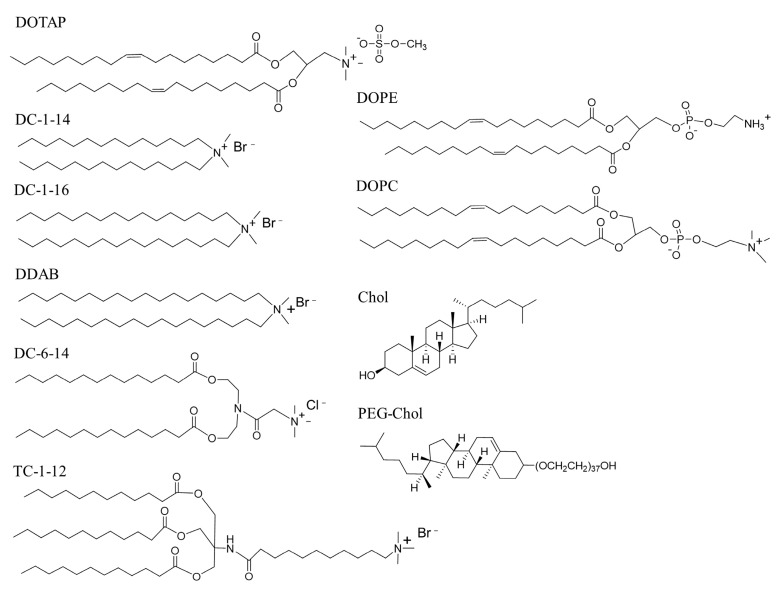
Structure of cationic lipids, neutral helper lipids, and the PEG-lipid used in this study. DOTAP: 1,2-dioleoyl-3-trimethylammonium-propane methyl sulfate salt; DC-1-14: *N*,*N*-dimethyl-*N*-tetradecyltetradecan-1-aminium bromide; DC-1-16: *N*-hexadecyl-*N*,*N*-dimethylhexadecan-1-aminium bromide; DDAB: *N*,*N*-dimethyl-*N*-octadecyloctadecan-1-aminium bromide; DC-6-14: 2-(bis(2-(tetradecanoyloxy)ethyl)amino)-*N*,*N*,*N*-trimethyl-2-oxoethan-1-aminium chloride; TC-1-12: 11-((1,3-bis(dodecanoyloxy)-2-((dodecanoyloxy)methyl)propan-2-yl)amino)-*N*,*N*,*N*-trimethyl-11-oxoundecan-1-aminium bromide; DOPE: 1,2-dioleoyl-*sn*-glycero-3-phosphoethanolamine; DOPC: 1,2-dioleoyl-*sn*-glycero-3-phosphocholine; Chol: cholesterol; PEG-Chol: polyethylene glycol-cholesteryl ether.

In this study, we prepared mRNA lipoplexes by mixing a lipid-ethanol solution with PBS containing mRNAs. To prepare the mRNA lipoplexes, we used DOTAP, DC-1-14, DC-1-16, DDAB, DC-6-14, and TC-1-12 as cationic lipids; DOPE, DOPC, and Chol as neutral helper lipids; and PEG-Chol as a dispersing agent (Figure 1), in a lipid-ethanol solution of cationic lipid/neutral helper lipid/PEG-Chol at a molar ratio of 49.5:49.5:1 (Table 1). For in vitro mRNA transfection using the MEI method, 100 μL PBS containing 0.5 μg mRNA was quickly added to the lipid-ethanol solution. The sizes of the LP-DOTAP/DOPC, LP-DC-1-14/DOPC, LP-DDAB/Chol, LP-DC-6-14/DOPE, and LP-DC-6-14/Chol lipoplexes were 284, 658, 265, 314, and 313 nm, respectively, and their PDIs were 0.25, 0.30, 0.14, 0.23, and 0.15, respectively (Table 2). In contrast, the other lipoplexes were in the range of 98–217 nm, with PDIs of 0.08–0.18.

### 3.2. In Vitro Protein Expression and Cell Viability after Transfection with mRNA Lipoplexes

**Figure 2:**
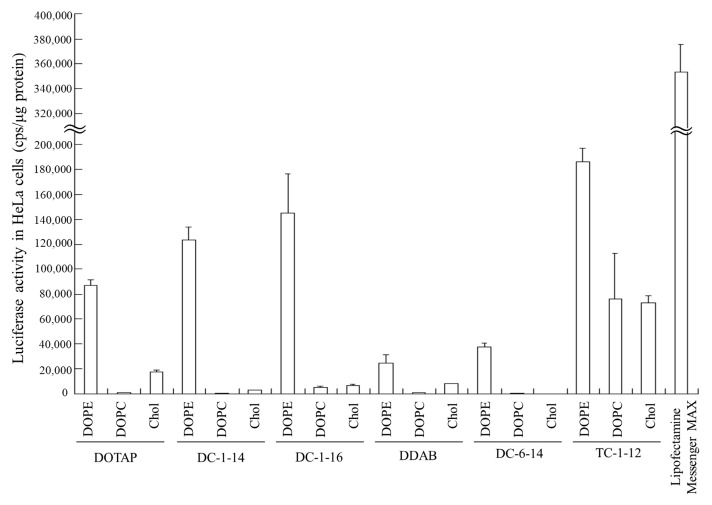
Effect of cationic and neutral helper lipids in mRNA lipoplexes on luciferase expression in HeLa cells after transfection with FLuc mRNA lipoplexes. mRNA lipoplexes with FLuc mRNA were added to HeLa cells at 0.5 μg/mL mRNA, and luciferase assays were carried out 24 h after incubation. Lipofectamine^®^ MessengerMAX^TM^ was used as a control for mRNA transfection. Each column represents the mean + S.D. (*n* = 3).

Luciferase activity was examined 24 h after transfection of FLuc mRNA lipoplexes into HeLa cells. Among all the mRNA lipoplexes, the LP-DC-1-14/DOPE, LP-DC-1-16/DOPE, and LP-TC-1-12/DOPE lipoplexes exhibited high luciferase activity (Figure 2). Commercially available Lipofectamine^®^ MessengerMAX^TM^ (Thermo Fisher Scientific, Inc.) exhibited approximately 2-fold higher luciferase activity than that in LP-TC-1-12/DOPE lipoplexes. Regardless of the cationic lipid type, the inclusion of DOPE in the liposomal formulation resulted in high luciferase activity, whereas the inclusion of DOPC or Chol led to low luciferase activity. No correlation was observed between mRNA lipoplex size (Table 2) and transfection activity (Figure 2). Therefore, DOPE was used as a neutral helper lipid in subsequent experiments. Among the mRNA lipoplexes used in this study, the LP-DC-1-14/DOPE, LP-DC-1-16/DOPE, and LP-TC-1-12/DOPE lipoplexes induced high luciferase expression in HeLa cells.

**Figure 3:**
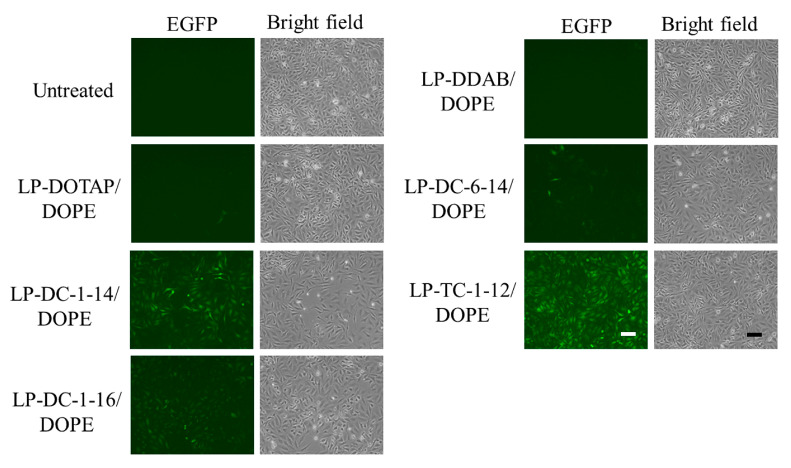
Effect of cationic lipids in mRNA lipoplexes on EGFP expression in HeLa cells after transfection with EGFP mRNA lipoplexes. mRNA lipoplexes with EGFP mRNA were added to HeLa cells at 0.5 μg/mL mRNA, and EGFP expression (green) was observed 24 h after incubation. Scale bar = 100 μm. EGFP: enhanced green fluorescent protein.

Next, we examined EGFP expression in HeLa cells 24 h after transfection with EGFP mRNA lipoplexes. The LP-DC-1-14/DOPE, LP-DC-1-16/DOPE, and LP-TC-1-12/DOPE lipoplexes exhibited high EGFP expression in HeLa cells (Figure 3), which was consistent with the luciferase expression results (Figure 2).

**Figure 4:**
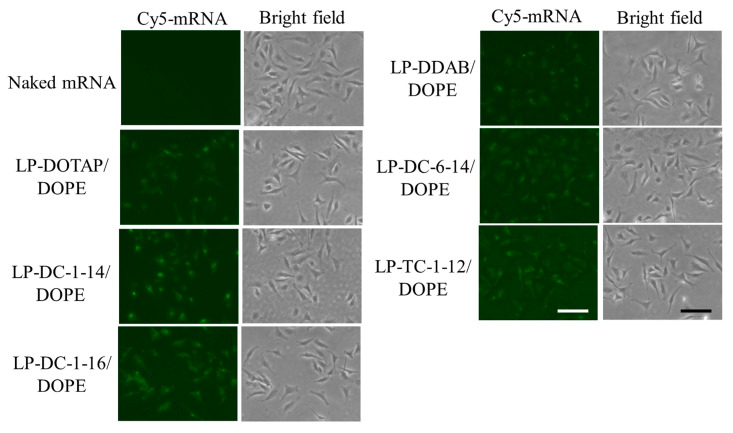
Effect of cationic lipids in mRNA lipoplexes on cellular uptake in HeLa cells after transfection with Cy5-mRNA lipoplexes. mRNA lipoplexes with Cy5-mRNA were added to HeLa cells at 0.5 μg/mL mRNA, and localization of Cy5-mRNA (green) was observed 3 h after incubation. Scale bar = 100 μm.

To understand why the LP-DC-1-14/DOPE, LP-DC-1-16/DOPE, and LP-TC-1-12/DOPE lipoplexes showed high protein expression levels, we investigated the cellular uptake of Cy5-mRNA 3 h after transfection. We detected more intense fluorescent signals for Cy5-mRNA in cells transfected with the LP-DC-1-14/DOPE, LP-DC-1-16/DOPE, and LP-TC-1-12/DOPE lipoplexes than in those transfected with the LP-DOTAP/DOPE, LP-DDAB/DOPE, or LP-DC-6-14/DOPE lipoplexes (Figure 4). This finding indicates that cationic lipids with short alkyl or acyl chain lengths (C12–C16) may efficiently internalize mRNA lipoplexes into cells compared to those with long alkyl or acyl chain lengths (C18). The mRNA levels in the cells closely corresponded to the efficacy of protein expression by the mRNA lipoplexes.

**Figure 5:**
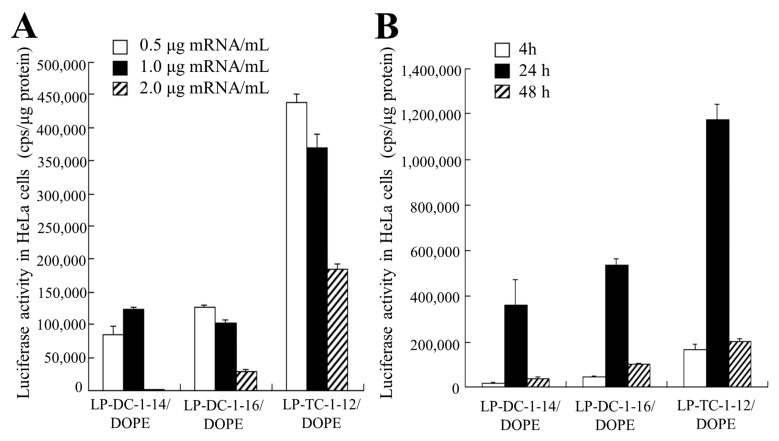
Effect of incubation time and mRNA concentration on luciferase expression in HeLa cells after transfection with FLuc mRNA lipoplexes. LP-DC-1-14/DOPE, LP-DC-1-16/DOPE, and LP-TC-1-12/DOPE lipoplexes with FLuc mRNA were added to HeLa cells. (**A**) Luciferase activities were measured 24 h after transfection of mRNA lipoplexes at 0.5, 1, or 2 μg/mL mRNA. (**B**) Luciferase activities were measured 4, 24, or 48 h after transfection of mRNA lipoplexes with FLuc mRNA at 0.5 μg/mL mRNA. Each column represents the mean + S.D. (*n* = 3).

We examined the effects of incubation time and mRNA concentration on protein expression in HeLa cells after transfection of LP-DC-1-14/DOPE, LP-DC-1-16/DOPE, and LP-TC-1-12/DOPE lipoplexes with FLuc mRNA. As a result, the highest luciferase activity by transfection with FLuc mRNA lipoplexes was observed at 0.5–1.0 mg/mL mRNA (Figure 5A) and after 24 h of transfection (Figure 5B).

**Figure 6:**
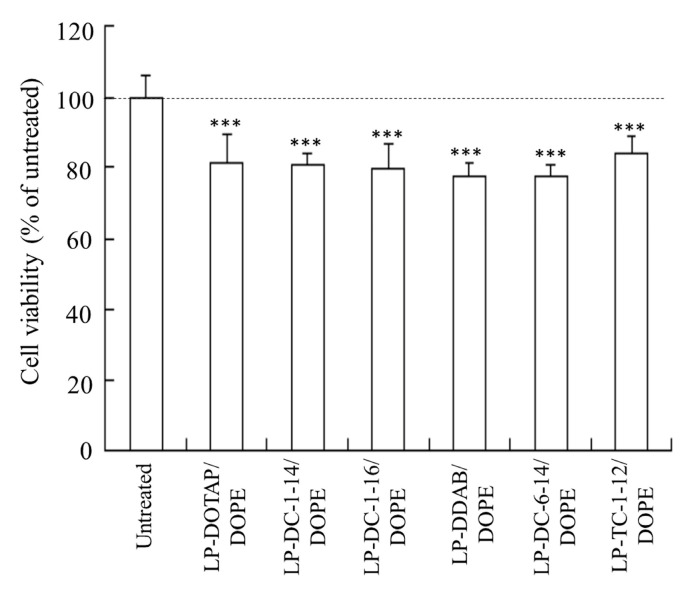
Effect of cationic lipids in mRNA lipoplexes on cell viability 24 h after transfection of FLuc mRNA lipoplexes into HeLa cells. FLuc mRNA lipoplexes were added to HeLa cells at 0.5 μg/mL mRNA. Each column represents the mean + S.D. (*n* = 6). *** *p* < 0.001, compared to untreated cells.

Furthermore, we investigated the viability of HeLa cells 24 h after transfection with mRNA lipoplexes (0.5 μg/mL). All lipoplexes exhibited slight cytotoxicity in HeLa cells (77–84% cell viability) (Figure 6).

**Figure 7:**
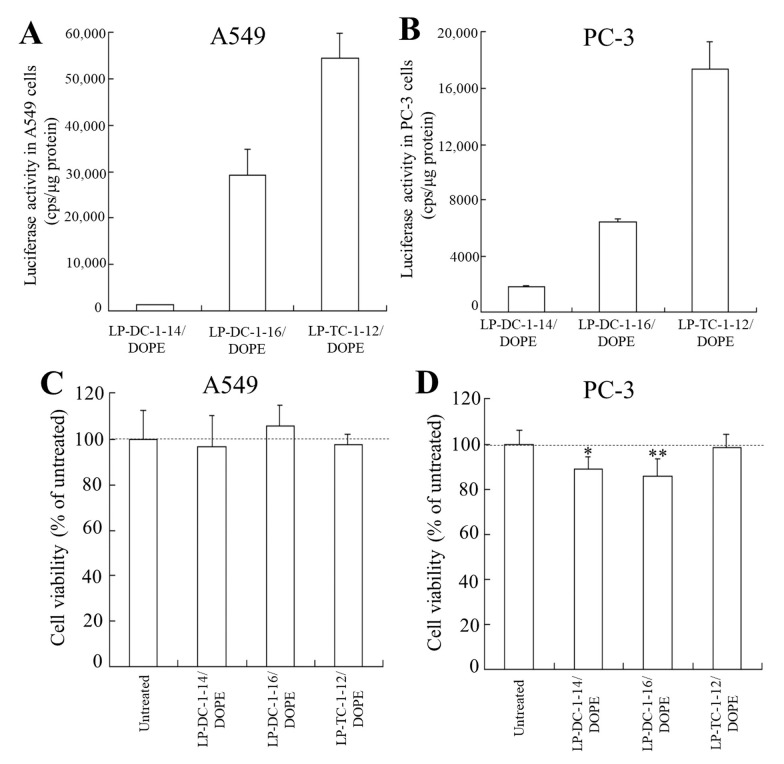
Effect of cell types on luciferase expression and viability in A549 and PC-3 cells after transfection with FLuc mRNA lipoplexes. LP-DC-1-14/DOPE, LP-DC-1-16/DOPE, and LP-TC-1-12/DOPE lipoplexes with FLuc mRNA were added to A549 (**A**,**C**) and PC-3 cells (**B**,**D**) at 0.5 μg/mL mRNA, and luciferase activity (**A**,**B**) and cell viability (**C**,**D**) were measured 24 h after incubation. Each column represents the mean + S.D. (*n* = 3 for A and B, *n* = 6 for C and D). * *p* < 0.05, ** *p* < 0.01, compared to untreated cells.

To examine the effect of the transfection activity of the mRNA lipoplexes in other cell lines, we transfected LP-DC-1-14/DOPE, LP-DC-1-16/DOPE, and LP-TC-1-12/DOPE lipoplexes into A549 and PC-3 cells. Among them, LP-DC-1-16/DOPE and LP-TC-1-12/DOPE lipoplexes exhibited high luciferase activity in both cell lines (Figure 7A,B), with no cytotoxicity in A549 cells (Figure 7C), while LP-DC-1-14/DOPE and LP-DC-1-16/DOPE lipoplexes showed slight cytotoxicity in PC-3 cells (89% and 86%, respectively) (Figure 7D). Altogether, the LP-DC-1-16/DOPE and LP-TC-1-12/DOPE lipoplexes induced high protein expression, regardless of the cell line into which they were transfected.

### 3.3. Biodistribution and Protein Expression of mRNA after Intramuscular Injection of mRNA Lipoplexes

**Figure 8:**
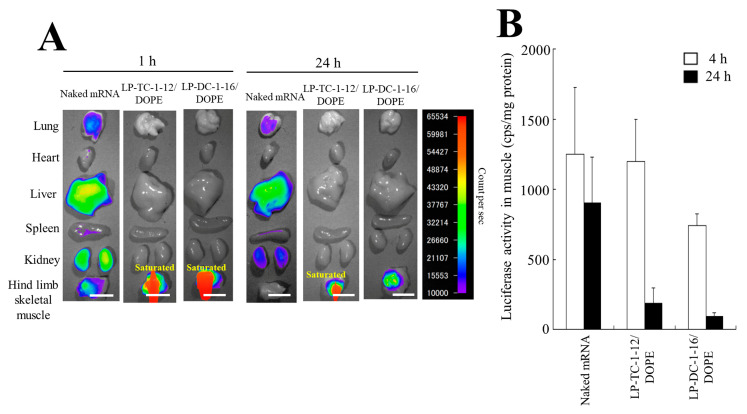
mRNA biodistribution and protein expression in mice after IM injection of mRNA lipoplexes. (**A**) mRNA lipoplexes with 5 μg of Cy5-mRNA were intramuscularly injected into mice. The mice were sacrificed 1 or 24 h after injection, and Cy5 fluorescence images of the tissues were acquired with an exposure time of 5 s. Scale bar = 1 cm (**B**) mRNA lipoplexes with 5 μg of FLuc mRNA (5moU) were administered intramuscularly into mice. The mice were sacrificed 4 or 24 h after injection, and the luciferase activity of the tissues was measured. Each column represents the mean + S.D. (*n* = 4–6).

We investigated the biodistribution of Cy5-mRNA 1 and 24 h after IM injection of mRNA lipoplexes into mice. The sizes of LP-DC-1-16/DOPE and LP-TC-1-12/DOPE lipoplexes prepared by the MEI method for in vivo transfection were 243 and 171 nm, respectively, and their PDIs were 0.20 and 0.12, respectively (Table 3). In addition, their ζ-potentials were approximately 28 and 36 mV, respectively. When LP-DC-1-16/DOPE and LP-TC-1-12/DOPE lipoplexes with Cy5-mRNA were injected into the hind limb skeletal muscle of mice, they exhibited high mRNA accumulation in the muscle at both time points after the injection (Figure 8A). In contrast, when naked Cy5-mRNA was injected into mice, accumulation of Cy5-mRNA was observed in the liver but not in the muscle at 24 h.

Furthermore, we analyzed luciferase expression in homogenates of skeletal muscles after IM injection of mRNA lipoplexes. However, IM injection of LP-DC-1-16/DOPE or LP-TC-1-12/DOPE lipoplexes with FLuc mRNA exhibited lower luciferase activity than the naked FLuc mRNA (Figure 8B), indicating that relatively large mRNA lipoplexes may not diffuse from the injection site (Figure S1), resulting in low protein expression.

### 3.4. Biodistribution and Protein Expression of mRNA after Systemic Injection of mRNA Lipoplexes

**Figure 9:**
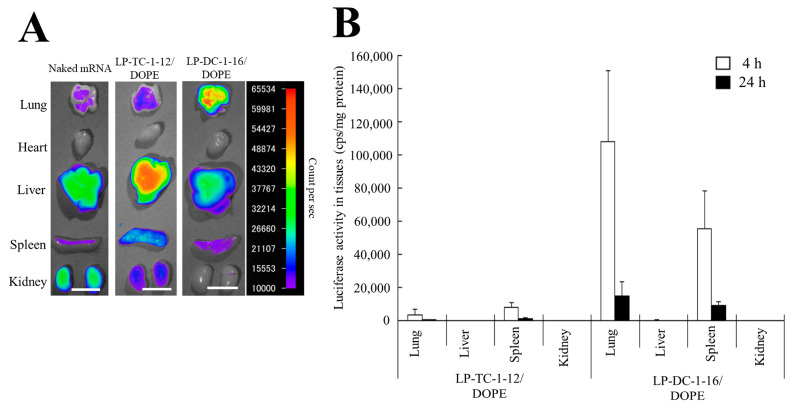
mRNA biodistribution and protein expression in mice after IV injection of mRNA lipoplexes. (**A**) mRNA lipoplexes with 10 μg of Cy5-mRNA were intravenously injected into mice. The mice were sacrificed 1 h after injection, and Cy5 fluorescence images of the tissues were acquired with an exposure time of 5 s. Scale bar = 1 cm. (**B**) mRNA lipoplexes with 20 μg of FLuc mRNA (5moU) were administered systemically into mice. The mice were sacrificed 4 or 24 h after injection, and the luciferase activity of the tissues was measured. Each column represents the mean + S.D. (*n* = 4–5).

We investigated the biodistribution of Cy5-mRNA 1 h after IV injection of mRNA lipoplexes into mice. When naked Cy5-mRNA and LP-TC-1-12/DOPE lipoplexes with Cy5-mRNA were systemically injected into mice, they exhibited high mRNA accumulation, mainly in the liver (Figure 9A). In contrast, IV injection of LP-DC-1-16/DOPE lipoplexes resulted in high mRNA accumulation in the lungs.

Furthermore, we analyzed luciferase expression in the homogenates of major organs after IV injection of mRNA lipoplexes. When LP-DC-1-16/DOPE lipoplexes with FLuc mRNA were systemically injected into mice, they exhibited high luciferase activity in the lungs and spleen, with maximum expression observed at 4 h post-administration (Figure 9B). In contrast, IV injection of LP-TC-1-12/DOPE lipoplexes resulted in lower luciferase activity than that of LP-DC-1-16/DOPE lipoplexes in the lungs and spleen. However, luciferase expression levels in the liver and kidney after IV injection of LP-DC-1-16/DOPE or LP-TC-1-12/DOPE lipoplexes were at the background level.

### 3.5. Induction of Anti-OVA Antibody by Intravenous Injections of OVA mRNA Lipoplexes

**Figure 10:**
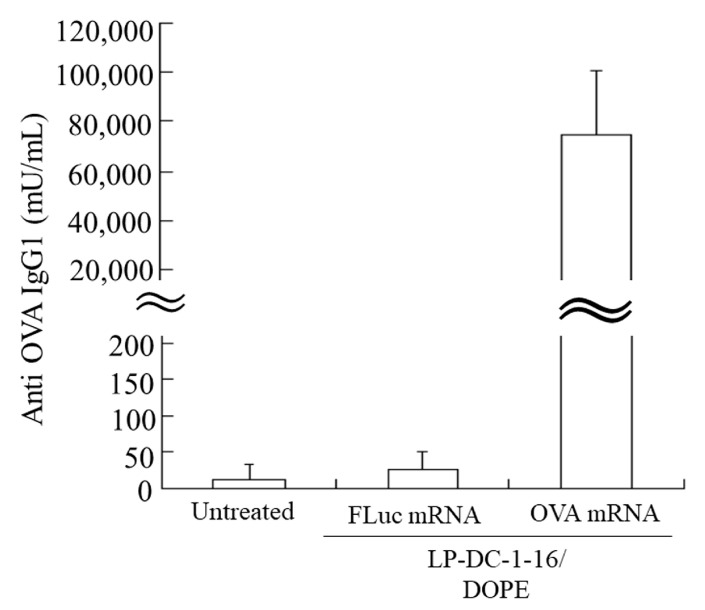
Induction of anti-OVA IgG1 after IV injection of OVA mRNA lipoplexes. LP-DC-1-16/DOPE lipoplexes with 20 μg FLuc mRNA (5moU) or OVA mRNA (5moU) were systemically injected into the mice on days 0 and 14. Blood was collected from immunized mice on day 28, and OVA-specific IgG1 in serum was quantified. Each column represents the mean + S.D. (*n* = 4). OVA: ovalbumin.

Finally, we used the LP-DC-1-16/DOPE lipoplexes to evaluate the induction of anti-OVA antibodies, as these lipoplexes exhibited higher luciferase expression in the spleen than the LP-TC-1-12/DOPE lipoplexes. The IgG1 response to OVA was measured two weeks after the second injection. No anti-OVA IgGs were detected in the sera of untreated mice and mice immunized with LP-DC-1-16/DOPE lipoplexes and FLuc mRNA (Figure 10). In contrast, IV injection of LP-DC-1-16/DOPE lipoplexes with OVA mRNA induced significantly higher anti-OVA IgG1 levels (approximately 75,000 mU/mL).

## 4. Discussion

In this study, we developed an MEI method for the preparation of mRNA lipoplexes and evaluated the in vitro and in vivo protein expression efficiencies. A liposomal formulation composed of cationic lipids containing unsaturated dialkyl (C14 or C16) chains (DC-1-14 and DC-1-16) or triacyl (C12) chains (TC-1-12) with DOPE enhanced the protein expression in cells. The inclusion of DC-1-14 or DC-1-16 in liposomal formulations resulted in higher protein expression in cells than those containing saturated and long dialkyl (DDAB) and unsaturated and long diacyl chains (DOTAP). These results indicate that cationic lipids with relatively short alkyl chain lengths may be suitable for the effective transfection of mRNA lipoplexes. In addition, Koulov et al. reported that cationic lipids with trialkyl chains enhanced vesicle fusion compared to those with structurally associated dialkyl or monoalkyl chains [30]. These findings indicate that TC-1-12 may have fusogenic activity and may be effective as a cationic lipid for mRNA delivery. The inclusion of the DOPE in the liposomal formulation caused strong protein expression compared to DOPC or Chol. This result is similar to that of a previous report stating that mRNA lipoplexes containing DOPE exhibited higher transfection efficiency than those containing DOPC [31]. DOPE may improve gene expression by destabilizing the endosomal membrane after the cellular uptake of mRNA lipoplexes via endocytosis.

In in vivo transfection, IM injection of LP-DC-1-16/DOPE and LP-TC-1-12/DOPE lipoplexes resulted in lower protein expression than that of naked mRNA (Figure 8B). After IM injection, mRNA lipoplexes remained at the injection site (Figure S1) and were not distributed in other tissues (Figure 8A). Di et al. reported that small LNPs (approximately 100 nm) spread more easily from the injection site than large particles (approximately 330 nm) when administered intramuscularly [17]. This implies that LP-DC-1-16 and LP-TC-1-12 lipoplexes cannot diffuse within tissues, resulting in low protein expression. Therefore, for IM injection, the mRNA lipoplexes should be smaller than 100 nm. Hence, further optimization of the IM injection of mRNA lipoplexes is required.

Systemic injection of LP-DC-1-16/DOPE lipoplexes resulted in high protein expression in the lungs and spleen, and induced an increase in OVA-specific IgG1 levels upon immunization. The spleen is the largest organ in the lymphatic system and produces an efficient immune response. This result indicates that OVA antigens expressed in the spleen may effectively induce the production of anti-OVA antibodies. In contrast, although the LP-TC-1-12/DOPE lipoplexes induced the highest protein expression in the spleen, the expression level was eight-fold lower than that of the LP-DC-1-16/DOPE lipoplexes. LP-TC-1-12/DOPE lipoplexes may be unstable in blood circulation following IV injection because of the short acyl chains of TC-1-12, resulting in accumulation in the liver and spleen. Chen et al. reported that cationic liposomes composed of DOTAP/DOPE/Chol at a molar ratio of 40:10:38 induce protein expression in the lungs, liver, and spleen, when lipoplexes containing FLuc mRNA are systemically injected [14]. Chol is often used to stabilize lipid bilayers. We did not evaluate whether the inclusion of Chol in the liposomal formulation of cationic lipids and DOPE increased the transfection efficiency in vivo; however, it might improve the in vivo stability of LP-TC-1-12/DOPE lipoplexes. Furthermore, the systemic injection of LP-TC-1-12/DOPE lipoplexes resulted in high mRNA accumulation, mainly in the liver (Figure 9A), but did not induce protein expression (Figure 9B), suggesting that mRNA accumulated in the liver might be degraded by RNase. Future experiments should evaluate the percentage of intact mRNA that accumulates in tissues. In addition, the hemolysis and stability of mRNA lipoplexes in blood circulation should be clarified in future studies. After in vitro transfection with mRNA lipoplexes, luciferase expression peaked at 24 h after transfection; however, in in vivo transfection, luciferase expression at 4 h after IV and IM injections was higher than at 24 h. Although the reason for this difference was not clear, this phenomenon was similar to a previous report [17]. Based on our study, LP-DC-1-16/DOPE lipoplexes could possibly be used to develop potential mRNA vaccines against viral infections or tumor immunity to achieve high efficacy.

An advantage of the MEI method for preparing mRNA lipoplexes is the production of homogeneous liposomes without sonication or dialysis [26]. In the present study, we prepared monodisperse mRNA lipoplexes (<0.20 PDI) for LP-DC-1-16/DOPE or LP-TC-1-12/DOPE by simply mixing lipid-ethanol solution and PBS containing mRNAs. In IV administration, the LP-DC-1-16/DOPE lipoplex suspension contained 14.3 μL ethanol per mouse. However, the 50% lethal dose (LD_50_) of ethanol for IV injections in mice with a 20 g body weight is 56 μL (2.8 mL/kg) [32]. Therefore, we believe that the MEI method used for the preparation of mRNA lipoplexes can be employed for in vivo mRNA transfection.

## 5. Conclusions

In this study, we developed an MEI method for mRNA transfection, in which injectable mRNA lipoplexes were prepared by simply mixing a lipid-ethanol solution with an mRNA solution. We found that the LP-DC-1-16/DOPE lipoplexes prepared using the MEI method exhibited high in vitro and in vivo protein expression. Thus, LP-DC-1-16/DOPE lipoplexes could possibly be used to develop mRNA vaccines for the prevention of infectious diseases and cancer immunotherapy. In addition, the MEI method can be widely applied to the production of mRNA lipoplexes.

## Figures and Tables

**Table 1:** Liposomal formulation for mRNA transfection with a lipid-ethanol solution.

Liposome	Formulation (mol%)
LP-DOTAP/DOPE	DOTAP/DOPE/PEG-Chol (49.5/49.5/1)
LP-DOTAP/DOPC	DOTAP/DOPC/PEG-Chol (49.5/49.5/1)
LP-DOTAP/Chol	DOTAP/Chol/PEG-Chol (49.5/49.5/1)
LP-DC-1-14/DOPE	DC-1-14/DOPE/PEG-Chol (49.5/49.5/1)
LP-DC-1-14/DOPC	DC-1-14/DOPC/PEG-Chol (49.5/49.5/1)
LP-DC-1-14/Chol	DC-1-14/Chol/PEG-Chol (49.5/49.5/1)
LP-DC-1-16/DOPE	DC-1-16/DOPE/PEG-Chol (49.5/49.5/1)
LP-DC-1-16/DOPC	DC-1-16/DOPC/PEG-Chol (49.5/49.5/1)
LP-DC-1-16/Chol	DC-1-16/Chol/PEG-Chol (49.5/49.5/1)
LP-DDAB/DOPE	DDAB/DOPE/PEG-Chol (49.5/49.5/1)
LP-DDAB/DOPC	DDAB/DOPC/PEG-Chol (49.5/49.5/1)
LP-DDAB/Chol	DDAB/Chol/PEG-Chol (49.5/49.5/1)
LP-DC-6-14/DOPE	DC-6-14/DOPE/PEG-Chol (49.5/49.5/1)
LP-DC-6-14/DOPC	DC-6-14/DOPC/PEG-Chol (49.5/49.5/1)
LP-DC-6-14/Chol	DC-6-14/Chol/PEG-Chol (49.5/49.5/1)
LP-TC-1-12/DOPE	TC-1-12/DOPE/PEG-Chol (49.5/49.5/1)
LP-TC-1-12/DOPC	TC-1-12/DOPC/PEG-Chol (49.5/49.5/1)
LP-TC-1-12/Chol	TC-1-12/Chol/PEG-Chol (49.5/49.5/1)

**Table 2:** Size of mRNA lipoplexes for in vitro transfection.

Liposome	Size (nm)	PDI
LP-DOTAP/DOPE	136.3 ± 0.1	0.09 ± 0.02
LP-DOTAP/DOPC	284.0 ± 7.1	0.25 ± 0.00
LP-DOTAP/Chol	112.1 ± 0.9	0.10 ± 0.02
LP-DC-1-14/DOPE	197.9 ± 0.5	0.08 ± 0.01
LP-DC-1-14/DOPC	657.9 ± 19.4	0.30 ± 0.01
LP-DC-1-14/Chol	178.2 ± 2.7	0.07 ± 0.02
LP-DC-1-16/DOPE	211.6 ± 4.3	0.05 ± 0.03
LP-DC-1-16/DOPC	156.9 ± 3.5	0.14 ± 0.01
LP-DC-1-16/Chol	196.5 ± 2.9	0.11 ± 0.02
LP-DDAB/DOPE	217.7 ± 4.0	0.08 ± 0.03
LP-DDAB/DOPC	120.9 ± 1.8	0.11 ± 0.02
LP-DDAB/Chol	265.1 ± 6.5	0.14 ± 0.01
LP-DC-6-14/DOPE	314.4 ± 5.4	0.23 ± 0.02
LP-DC-6-14/DOPC	207.7 ± 1.2	0.18 ± 0.01
LP-DC-6-14/Chol	313.4 ± 6.7	0.15 ± 0.02
LP-TC-1-12/DOPE	137.0 ± 2.5	0.12 ± 0.01
LP-TC-1-12/DOPC	97.6 ± 1.3	0.09 ± 0.01
LP-TC-1-12/Chol	133.7 ± 0.4	0.14 ± 0.01

Values represent means ± S.D. (*n* = 3). PDI: polydispersity index.

**Table 3:** Size and ζ-potential of mRNA lipoplexes for in vivo transfection.

Liposome	Size (nm)	PDI	ζ-Potential (mV)
LP-DC-1-16/DOPE	243.4 ± 1.3	0.20 ± 0.01	28.3 ± 0.9
LP-TC-1-12/DOPE	170.8 ± 0.9	0.12 ± 0.01	35.9 ± 0.7

Values represent means ± S.D. (*n* = 3). PDI: polydispersity index.

## Data Availability

Not applicable.

## Supplementary Materials

The following are available online at https://www.mdpi.com/article/10.3390/pharmaceutics15041141/s1, Figure S1: Localization of mRNA in mice after intramuscular injection of mRNA lipoplexes. mRNA lipoplexes with 5 μg of Cy5-mRNA were administered intramuscularly into mice. One or twenty-four hours after injection, tissue was frozen and sliced to observe localization of Cy5-mRNA (green) using a fluorescent microscope. Scale bar = 100 μm.
