# Supplementary material for: Efficient mRNA Delivery with mRNA Lipoplexes Prepared Using a Modified Ethanol Injection Method
Source: Pharmaceutics. 2023 Apr 4;15(4):1141. doi: 10.3390/pharmaceutics15041141 (PMC10146866; doi:10.3390/pharmaceutics15041141)
Supplement: Supplementary file 1 [file pharmaceutics-15-01141-s001.zip › pharmaceutics-2285063-supplementary.pdf]

Supplementary Data

Efficient mRNA Delivery with mRNA Lipoplexes Prepared using a Modified Ethanol Injection Method

Min Tang, Ayane Sagawa, Nodoka Inoue, Satomi Torii, Kana Tomita and Yoshiyuki Hattori*

Department of Molecular Pharmaceutics, Hoshi University, 2-4-41 Ebara, Shinagawa, Tokyo 142-8501, Japan

Correspondence to: Professor Yoshiyuki Hattori, Department of Molecular Pharmaceutics, Hoshi University, 2-4-41, Ebara, Shinagawa-ku, Tokyo 142-8501, Japan
E-mail: yhattori@hoshi.ac.jp

Materials and methods

Localization of Cy5-mRNA after intramuscular injection of mRNA lipoplexes into mice

mRNA lipoplexes, with 5 μg of CleanCap® Cy5-mRNA, were intramuscularly administered into mice. One and twenty-four hours after intramuscular injection, tissue samples were frozen on dry ice and sliced into 16 μm sections. The localization of Cy5-mRNA was examined using a fluorescent microscope (Eclipse TS100-F; Nikon Corporation).

Results

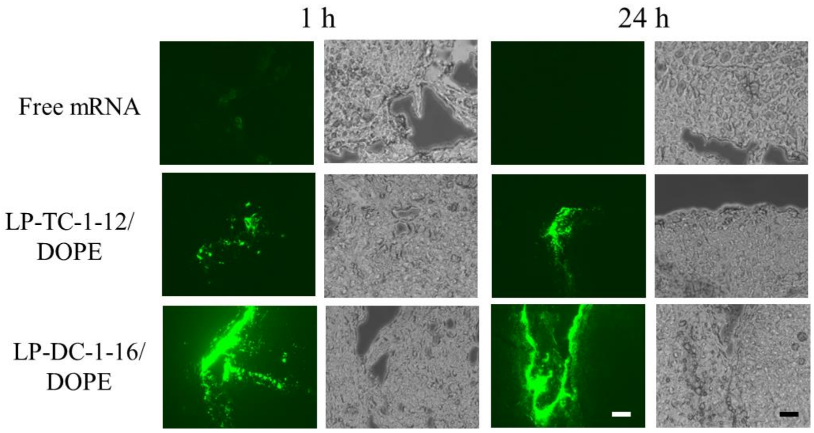

Figure S1 Localization of mRNA in mice after intramuscular injection of mRNA lipoplexes. mRNA lipoplexes with 5 μg of Cy5-mRNA were administered intramuscularly into mice. One or twenty-four hours after injection, tissue was frozen and sliced to observe localization of Cy5-mRNA (green) using a fluorescent microscope. Scale bar = 100 μm.
